# Persistent Shoulder Pain After Anterior Cervical Discectomy and Fusion (ACDF): Another Dual Pathology

**DOI:** 10.7759/cureus.13709

**Published:** 2021-03-05

**Authors:** Shahbaz Khan, Nida Hameed, Saddam Mazar, Imtiaz A Hashmi, Mohammad S Rafi, Mohammad Idrees Shah, Nadeem A Baloch

**Affiliations:** 1 Orthopedics and Spine Surgery, Ziauddin University Hospital, Karachi, PAK; 2 Orthopedics and Traumatology, Dr. Ziauddin Hospital, Karachi, PAK; 3 Orthopedic Surgery, Dr. Ziauddin Hospital, Karachi, PAK; 4 Orthopedics/Spine and Orthopedic Surgery, Agha Khan University Hospital, Karachi, PAK; 5 Orthopedics/Spine and Orthopedic Surgery, Dr. Ziauddin University Hospital, Karachi, PAK; 6 Orthopedics, Dr. Ziauddin University Hospital, Karachi, PAK; 7 Orthopedics, Dr. Ziauddin Hospital, Kemari Campus, Karachi, PAK

**Keywords:** dual pathologies, acdf, rotator cuff tears

## Abstract

Purpose

It is often difficult for the clinician to isolate the etiology of pain occurring either in the neck or shoulder because of the reason that neck pain can refer to the shoulder and vice versa. Concordance research has found that around one in 10 patients who were referred for cervical radiculopathy had comorbid shoulder pathology. The goal of this research is to analyze and correlate risk factors for persistent shoulder pain (non-dermatomal) following cervical spine surgery.

Methods

This was a single-center, retrospective study. The medical records of patients admitted for anterior cervical discectomy and fusion (ACDF) were reviewed from August 2018 to Feb 2021. Patients of both sexes and age more than 18 years who underwent ACDF (single/multiple levels) were included and the medical record was checked for whether they had persistent shoulder pain following ACDF. The proportion of patients undergoing shoulder surgery for associated rotator cuff tears and subacromial impingement were recorded.

Results

Seventy patients presenting with cervical prolapsed intervertebral disc (PID) were studied. A majority of our patients were females (n=48, 68.6%) and males (n=22, 31.4%) with an M:F ratio of 1:2 and the majority of patients were between the ages of 40 to 60 years (n=34, 48.6%). After surgical intervention (ACDF), 48 patients (68.6%) noted the cessation of shoulder symptoms (pain, weakness, and numbness) during their last visit. Rotator cuff tear (supraspinatus mainly) was the predominant finding in MRI in those who didn’t improve after ACDF (n=18, 25.7%, p-value: 0.001). Twenty patients (28.6%) underwent acromioplasty and rotator cuff tendon repair and four patients responded well to subacromial injection. The C6-7 level was most commonly affected (n=48, 68.6%) followed by C5-6 level (n=19, 27.1%). No significant association was found between cervical levels with shoulder pathologies (p-0.171), though a significant association between a visual analog scale (VAS) score >7 after surgery with shoulder pathologies (p-0.001) was found. The C6-7 level was commonly affected in females (p=0.038) but no significant association between gender and shoulder pathologies was found (p=0.332).

Conclusion

Dual pathologies in patients with cervical PID are very common. It needs careful attention by doing thorough clinical examination and correlating patient symptoms with radiological investigations. A patient who presents with persistent shoulder pain after cervical spine surgery had a higher chance of having concurrent shoulder pathology, and they should be properly investigated and managed to alleviate the suffering of the patient.

## Introduction

It may be difficult for the clinician to isolate the etiology of pain occurring either in the neck or shoulder because neck pain can refer to the shoulder and vice versa [1]. Furthermore, neck and shoulder pathology also coexist, creating a care issue to be tackled first [2]. One of the most prominent causes of posterior neck and shoulder pain is cervical spondylosis [3]. However, the true cause of a patient's symptoms can be identified and handled with a detailed history, clinical examination, and imaging studies [4].

Painful impingement of the shoulder is reported to occur in up to 24% of cervical radiculopathy patients [5]. Concordance research found that around one in 10 patients who were referred for cervical radiculopathy had comorbid shoulder pathology [5]. To differentiate whether rotator cuff or cervical spine or both are culprits, it is essential to do mapping of pain (site, onset, radiation (up to the elbow or beyond the elbow), numbness, night pain, relived on shoulder abduction or not), relevant clinical (non-invasive impingement signs) and invasive (subacromial injection) tests and neuro-radiological evaluation, which includes shoulder and cervical spine radiographs and screening MRI. 

Cervical spine decompression surgery (anterior/posterior) is performed to maximize neurological recovery and minimize pain to achieve good functional outcomes in patients with radiculopathy. Persistent pain after cervical spine surgery, however, is not unusual [6]. In these dual pathology cases, it is often troublesome to decide what to address first, both surgeries simultaneously, or cervical first followed by shoulder, or vice versa.

The principal goals of this study are to: (1) Correlate the level of the cervical spine with shoulder pathologies (primary goal); (2) Analyze and assess the risk factors of chronic shoulder pain following surgery on the cervical spine; (3) Understand whether anterior or posterior cervical decompression is associated with shoulder pain.

Generally, it is thought that lower cervical levels and posterior cervical decompression surgeries carry a risk of significant chronic shoulder pain.

## Materials and methods

This was a single-center, retrospective study. The medical records of patients admitted for anterior cervical discectomy and fusion (with severe radicular pain and failed conservative trial of collar and pain management) were reviewed from August 2018 to Feb 2021. Patients of both sex, of age more than 18 years who underwent ACDF (single/multiple levels), were included and the medical record was checked for whether they had persistent shoulder pain (non-dermatomal) following ACDF. On initial presentation, in case of a query, a subacromial diagnostic injection of xylocaine 2% was also used to differentiate pain due to shoulder pathology from radicular pain because of the cervical prolapsed intervertebral disc (PID). The proportion of patients having persistent non-dermatomal pain in the shoulder were further evaluated with MRI (if not done previously) and the diagnosis of dual pathology (rotator cuff tear, etc.) was made. The proportion of patients undergoing shoulder surgery for associated rotator cuff tears and subacromial impingement was recorded. A clinicoradiological correlation of patients was performed by two orthopedic and spine surgeons, at different times, in an unbiased manner. Patients with a significant history of trauma to the shoulder in the past, patients with inflammatory pathologies like rheumatoid arthritis, etc., and skeletally immature patients, with no radiological investigation available were excluded from the study.

Medical records were reviewed and data on age, sex, level of cervical spine surgery, the approach of the cervical spine (anterior in our series, as no trauma cases were included), visual analog score (VAS) before and after ACDF, type of shoulder pathology, and subsequent treatment received were recorded on pre-designed proforma. For comparisons of categorical variables, Fisher’s exact test was used and continuous variables were compared either using student’s t-test after verification of normal distribution by the Kolmogorov-Smirnov test or using the non-parametric Mann Whitney U-test. A p-value of less than 0.05 was regarded to be statistically significant.

## Results

**Figure 1:**
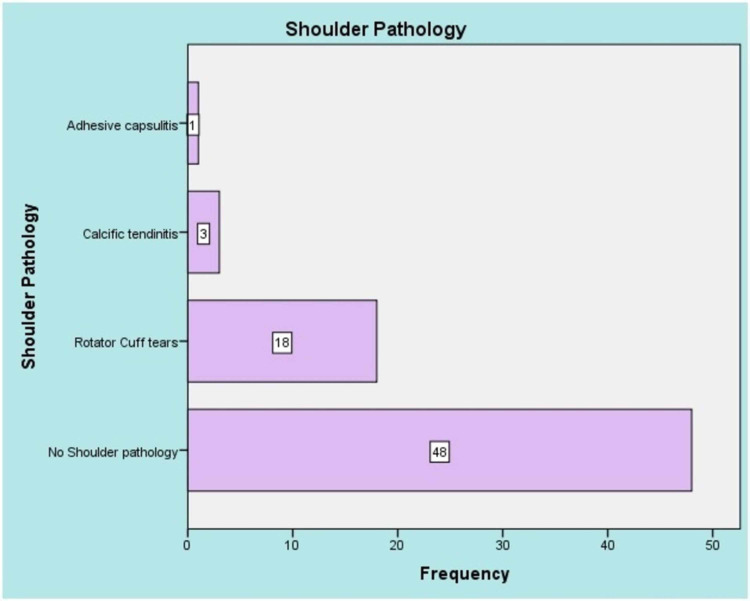
Type of shoulder pathologies

Overall, the medical records of 70 patients with dual pathologies in the cervical spine and shoulder undergoing operative management were reviewed. The majority of our patients were females (n=48, 68.6%), males were n=22, 31.4%, and between the ages of 40 to 60 years (n=34, 48.6%) (Table 1). After surgical intervention (ACDF), 48 patients (68.6%) noted the cessation of shoulder symptoms (pain, weakness, and numbness) during their last visit. Rotator cuff tear (supraspinatus mainly) was the predominant finding in those who didn’t improve after ACDF (n=18, 25.7%) (Figure 1). Twenty patients (28.6%) underwent acromioplasty and rotator cuff tendon repair, and four patients responded well to a subacromial injection. The C6-7 level was most commonly affected (n=48, 68.6%) followed by the C56 level (n=19, 27.1%). No significant association was found between the lower cervical levels with shoulder pathologies (p-0.171) (Table 2) There was a significant association between VAS score >7 after surgery with shoulder pathologies (p-0.001). The C6-7 level was commonly affected in females (p-0.038) and no significant association between gender and shoulder pathologies was found (p-0.332).

**Table 1:** Patient characteristics

	Age Groups	(n)	Percentage
	18-30.	3	4.3
Age	30--40	25	35.7
	40-60	34	48.6
	>60	8	11.4
	Total	70	100
	Male	22	31.4
Gender	Female	48	68.6
	Total	70	100
Level	C5-6	19	27.1
	C6-7	48	68.6
	Two level	3	4.3
	Total	70	100

**Table 2:** Correlation of gender with level of cervical spine and shoulder pathologies RCT: rotator cuff tears

		Level of Cervical Spine			
		C5-6	C6-7	Total	p-value
Sex	Male	2	19	22	
	Female	17	29	48	0.038
	Total	19	48	70	
			Shoulder Pathology		
		No pathology	RCT	Adhesive capsulitis	
Sex	Male	14	7	1	
	Female	34	11	0	0.332
	Total	48	18	1	
		No Shoulder pathology	RCT	Adhesive capsulitis	
Level Affected	C5-6	16	3	0	
	C6-7	29	15	1	0.171
	Two level	3	0	0	
	Total	48	18	1	

## Discussion

It can also be a lengthy and complex clinical path of shoulder pain following cervical spine surgery, often resulting in either multiple operations or even chronic symptoms. Proper workup, including thorough clinical examination and radiological investigations (MRI shoulder) should be done as part of screening if any clue is found on clinical examination. Confirmation of the source of the pain can be withdrawn by putting a subacromial local anesthetic injection; if the pain improves, the shoulder is the source rather than the cervical spine [7]. In the present study, the most common level affected was C6-7 (48, 68.6%) followed by the C56 level (19, 27.1%) as compared to the study by Gore et al. [8]. Studies have demonstrated a correlation between cervical and lumbar pathologies with rotator cuff tears according to age group (<60 years-13% rotator cuff tear (RCT), >60 years-25% RCT) [9].

Zhang et al. addressed the potential temporal association between cervical pathologies with RCT [10]. They found that 7% of patients who have cervical spine pathology would develop new RCT pathology or will undergo shoulder surgery for RCT in five years, possibly due to worsening muscle atrophy and degeneration associated with denervation from a chronic cervical spine disease [10]. Hattrup et al. performed a review on the association of RCT with cervical spine pathologies [1]. They proposed that biomechanical connections linking dual pathology can be mediated by the irritation or dysfunction of axillary and suprascapular nerves, both of which carry fibers from the C5 and C6 roots.

In the present study, we observed that patients with RCT experience more pain at night while turning on the affected side, as night-time pain indicates a chronic inflammatory process with an increase in blood flow. Though all tears are not painful, it mainly depends on the size of the tear [11]. Studies reported the relationship between the surgical approach in the cervical spine and its risk factors for axial neck pain [6]. They conclude that the posterior surgical approach is a risk factor for aggravating axial neck pain.

The current research involves patients who have undergone cervical surgery without having previously reported evidence of concurrent preexisting shoulder pathology. There is no consensus to date on the treatment of patients with both cervical and shoulder diseases. We conclude that due to the agonizing and dominant symptoms of cervical pathology, the shoulder pathology was an oversight. Thorough investigations should be performed to reach the origin of pain, which can arise from the spine, shoulder, or both, for proper strategic surgical planning. If the shoulder is the primary cause, it should be managed first followed by careful observation of cervical spine pathology and vice versa. For complex patients with equal contribution from shoulder and cervical spine, surgery should be planned for both either simultaneously or planned soon after the first surgery to mitigate the suffering of the patient and improve rehabilitation.

The limitations of our study include its retrospective study design and relatively small sample size.

## Conclusions

Our finding revealed that when spine surgeons experience lower cervical spine disorder with shoulder pain, the history and physical evaluation of the shoulder joint should carefully determine the concomitant shoulder disorder. Patients who present with persistent shoulder pain after cervical spine surgery have a higher chance of having concurrent shoulder pathology, and they should be properly investigated and managed to alleviate suffering.
